# Comprehensive profiling and quantitation of oncogenic mutations in non small-cell lung carcinoma using single molecule amplification and re-sequencing technology

**DOI:** 10.18632/oncotarget.10464

**Published:** 2016-07-07

**Authors:** Shirong Zhang, Bing Xia, Hong Jiang, Limin Wang, Rujun Xu, Yanbin Shi, Jianguang Zhang, Mengnan Xu, David S. Cram, Shenglin Ma

**Affiliations:** ^1^ Department of Oncology, Hangzhou First People's Hospital, Nanjing Medical University, Zhejiang, Hangzhou 310006, China; ^2^ Berry Genomics Corporation, Beijing 100015, China

**Keywords:** non small-cell lung carcinoma, oncogenic mutations, single molecule amplification and re-sequencing technology, allele-specific amplification refractory mutation system

## Abstract

Activating and resistance mutations in the tyrosine kinase domain of several oncogenes are frequently associated with non-small cell lung carcinoma (NSCLC). In this study we assessed the frequency, type and abundance of EGFR, KRAS, BRAF, TP53 and ALK mutations in tumour specimens from 184 patients with early and late stage disease using single molecule amplification and re-sequencing technology (SMART). Based on modelling of EGFR mutations, the detection sensitivity of the SMART assay was at least 0.1%. Benchmarking EGFR mutation detection against the gold standard ARMS-PCR assay, SMART assay had a sensitivity and specificity of 98.7% and 99.0%. Amongst the 184 samples, EGFR mutations were the most prevalent (59.9%), followed by KRAS (16.9%), TP53 (12.7%), EML4-ALK fusions (6.3%) and BRAF (4.2%) mutations. The abundance and types of mutations in tumour specimens were extremely heterogeneous, involving either monoclonal (51.6%) or polyclonal (12.6%) mutation events. At the clinical level, although the spectrum of tumour mutation(s) was unique to each patient, the overall patterns in early or advanced stage disease were relatively similar. Based on these findings, we propose that personalized profiling and quantitation of clinically significant oncogenic mutations will allow better classification of patients according to tumour characteristics and provide clinicians with important ancillary information for treatment decision-making.

## INTRODUCTION

Genetic profiling of tumour specimens from patients with non small-cell lung carcinoma (NSCLC) has identified clinically significant genetic variants in several oncogenes [1–4]. It has been postulated that these mutation events acts a key drivers of abnormal tumour growth and differentiation [5–6]. The most common mutations associated with NSCLC are found in the epithelial growth factor receptor (*EGFR*) gene. In unselected patients, 10-50% of tumour biopsies are EGFR mutation positive with L858R and exon 19 deletions in the tyrosine kinase domain accounting for up to 90% of all mutations [7]. Both mutation types result in an increase in constitutive tyrosine kinase activity and are hypersensitive to the reversible action of the small molecule tyrosine kinase inhibitors (TKIs) [8–11]. Conversely, two exon 20 mutations T790M [12, 13] and E20 insertions [14, 15] confer resistance to the action of TKIs on activating mutations by inducing a conformation change that re-activates the tyrosine kinase domain.

Apart from EGFR mutations, EML4-ALK fusions proteins are also found in 2-13% of NSCLC patients [16–18] who generally show a dramatic and prolonged response to TKI therapy [19, 20]. Both activating and resistance mutations in other oncogenes such as *KRAS* and *BRAF* [21–23] as well as inactivating mutations in the tumour suppressor gene *TP53* [24–26] are also found in NSCLC patients. Recent genome wide profiling has also identified sporadic mutations in other oncogenes, including *AKT1, FGFR3, HER2, MAP2K1, cMET, MEK, HRAS, NRAS, PIK3CA, ROS1* and *RET1* [21, 22, 27, 28], which are found in a low, but significant percentage of NSCLC patients and, present new potential drug targets.

The application of PCR and next generation sequencing (NGS) based diagnostic methods has proven to be relatively more sensitive and specific for detection of oncogenic mutations in formalin fixed biopsy samples [21, 23, 26, 29–31]. Recently, oncogene kits based on allele-specific amplification refractory mutation system PCR (ARMS-PCR) have been approved by the Chinese FDA for clinical diagnosis of oncogenic NSCLC mutations and are now routinely used in Chinese hospital diagnostic laboratories [32]. However, for the diagnosis of EML4-ALK gene rearrangements, fluorescent in situ hybridization (FISH) using specific *ALK* gene rearrangement probes, remains the gold standard diagnostic methodology [16]. Given the diversity of oncogenic mutations associated with NSCLC tumours, a more comprehensive methodology with the capacity to simultaneously detect and quantitate multiple oncogenic mutations in a single assay format is urgently needed to more accurately evaluate the patient's tumour mutation profile and identify which subgroups of patients may benefit from targeted drug therapies [33].

In this study, we evaluate the performance of a recently developed single allelic molecule counting methodology termed single molecule amplification and re-sequencing technology (SMART) [34] for the purpose of detecting and quantitating hot spot EGFR, KRAS, BRAF, ALK and TP53 mutations in NSCLC tumour specimens and define mutation profiles of early and advanced stage disease.

## RESULTS

### NSCLC patients and study design

The baseline clinical characteristics of the 184 study participants are summarized in Table 1. The median age of the patient cohort was 61.0 years, with the majority (116, 63%) under the age of 65 years old. The ratio of male to female subjects was 109:75. In relation to smoking history, 99 patients (53.8%) reported either a current or previous history of smoking. The majority of patients (156, 84.8%) had histologic subtypes of adenocarcinoma whereas a minority had other histologic subtypes, comprising squamous carcinoma (24, 13%), neuroendocrine carcinoma (2, 1.1%) and mucoepidermoid carcinoma (2, 1.1%). Applying clinical disease classification criteria, there were 63 stage I (34.2%), 26 stage II (14.1%), 27 stage III (14.7%) and 68 stage IV (37.0%) patients. Based on patient demographics and clinical parameters, the study cohort was representative of a fairly typical group of patients with diverse pathologies and stages of NSCLC.

**Table 1:** Patient demographics and clinical information versus EGFR mutation status

Clinical variables	No of patients (%)	EGFR mutation status by SMART assay		Significance (P value)
		Mutation positive	Mutation negative	
Age				
≤ 65	116 (63.0%)	52	64	0.2325 (> 0.05)
>65	68 (37.0%)	28	40	
Gender				
Male	109 (59.2%)	37	72	0.0017 (< 0.05)
Female	75 (40.8%)	43	32	
Smoking history				
Never	85 (46.2%)	49	36	0.0003 (< 0.05)
Ever	99 (53.8%)	31	68	
Histology #				
Adenocarcinoma	156 (86.7%)	74	82	0.094 (> 0.05)
Squamous carcinoma	24 (13.3%)	7	17	
Stage of NSCLC				
Early (I+II)	89 (48.4%)	37	52	0.6138 (> 0.05)
Late (III+IV)	95 (51.6%)	43	52	

# The two tumours with mucoepidermoid carcinoma and two with neuroendocrine carcinoma were excluded from the analysis

**Figure 1:**
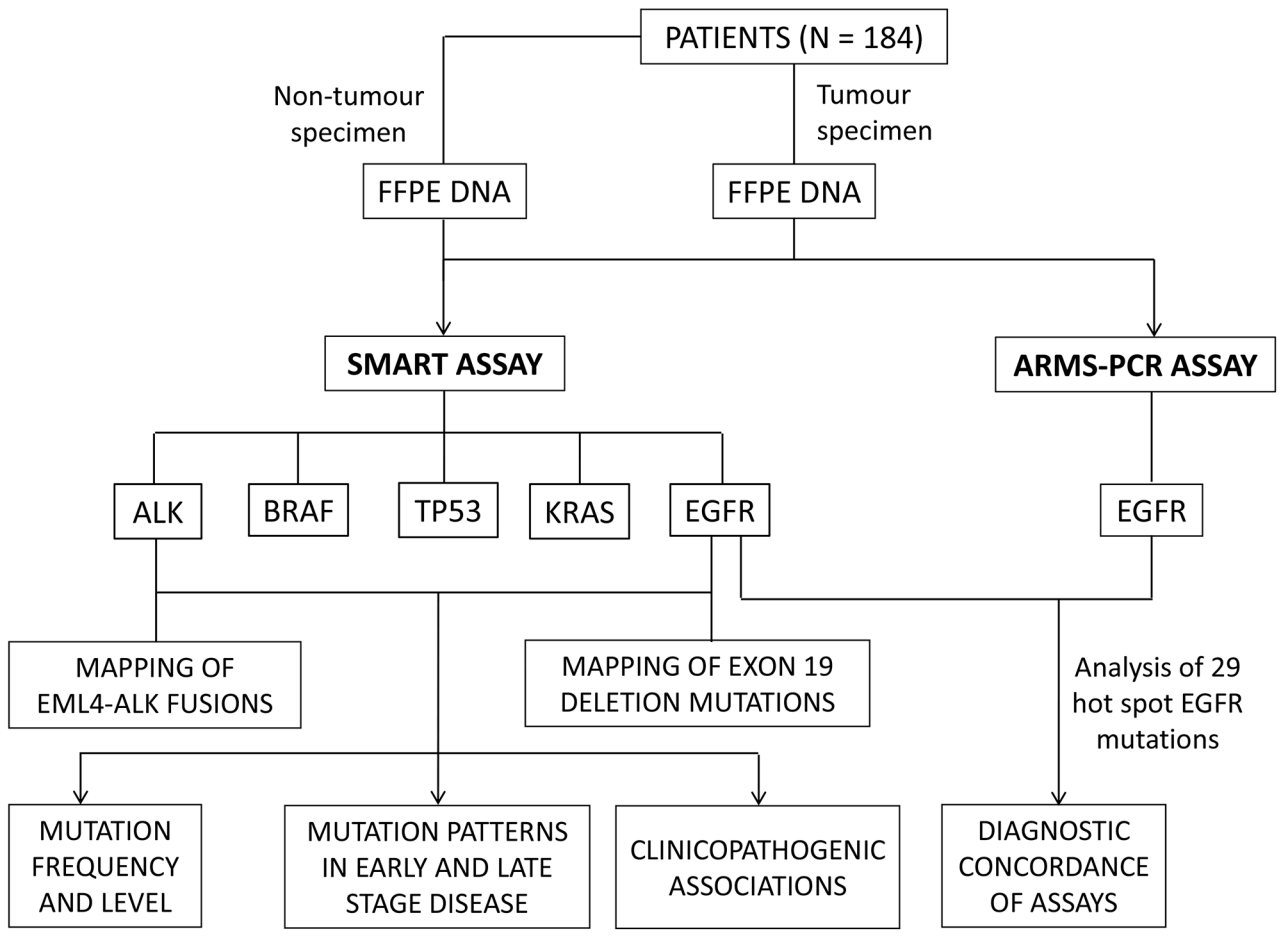
Study design

The overall study design is shown in Figure 1. Formalin fixed tumour specimens collected from 184 naive NSCLC patients were analysed independently by the ARMS-PCR and SMART assays for the presence of EGFR mutations. The SMART assay was also designed to simultaneously detect KRAS, TP53, BRAF and ALK fusion mutations (Supplementary Table S1). A full summary of mutation positive and negative patients is presented in Supplementary Tables S2 and S3, respectively.

### Validation of SMART assay for quantitation of EGFR mutations

**Figure 2:**
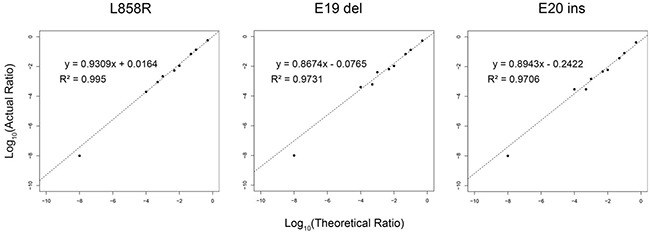
Sensitivity of SMART assay for detection of common EGFR variants A single nucleotide substitution L858R, an exon 19 deletion (746-750) and the exon 20 insertion (V774insH) were modelled at levels of 0.01%, 0.05%, 0.1%, 0.5%, 1%, 5%, 10% and 50%. By Pearson correlation, there was a linear relationship between actual (Y-axis) and theoretical log_10_ values (X-axis).

The sensitivity of the SMART assay to quantitate EGFR mutations was evaluated by creating three artificial models of the exon 21 point mutation L858R, the exon 19 deletion (del746-750) and the exon 20 insertion (V774insH) with increasing mutation input levels of 0.01%, 0.05%, 0.1%, 0.5%, 1%, 5%, 10% and 50%. By Pearson correlation (Figure 2), there was a linear relationship between observed and expected mutation levels, with R^2^ values of 0.995 (L858R), 0.973 (E19 del746-750) and 0.971 (E20 V774insH), indicating that the SMART assay was quantitative over a wide dynamic range of test mutation levels, with a detection sensitivity of 0.01%. For mutation analysis of 184 NSCLC tumour biopsy samples using the SMART assay, we conservatively opted for a mutation level cut-off of > 0.1%, equivalent to one mutant allele in 1,000 alleles, as the positive mutation detection threshold.

**Figure 3:**
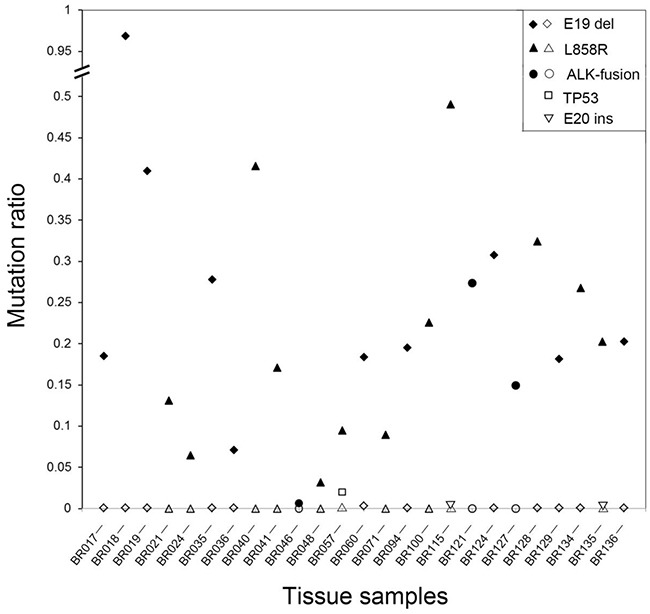
Levels of oncogenic mutations in matching tumour and non-tumour specimens A total of 25 patients were studied with either activating EGFR or ALK mutations identified in the original tumour specimens. All non-tumour tissues were negative but low levels of new mutations were identified in four cases. Closed symbols represent the level of activating mutation in the tumour tissue whereas open symbols represent the level of activating or new mutations in the non-tumour tissue.

The specificity of the multiplex SMART assay was also evaluated by comparing the profiles of mutation positive tumours with the corresponding adjacent non-tumour tissue (physical distance of at least 2 cm to tumor tissue) serving as a control (Figure 3, Supplementary Table S4). All control tissues were verified as histologically normal lung tissue by two pathologists. A total of 25 tumour tissues with activating EGFR mutations L858R (n=12) and E19 deletions (n=10) as well as activating ALK fusions (n=3) and matching control tissue were selected for comparative analysis. In all 25 cases, the non-tumour tissue was negative for the original tumour mutations (Figure 3), indicating high specificity of the assay. Interestingly, in four of the 25 non-tumour samples, a very low level of new mutations were identified, including one sample with the R248W TP53 mutation (CBR057, mutation ratio of 2.02%), one sample with a different EGFR E19 deletion variant (CBR060, mutation ratio of 0.26%) and two samples with EGFR E20 insertion variants (CBR115 and CBR135, mutation ratios of 0.06% and 0.04%, respectively).

### Concordance of SMART and ARMS-PCR assays for EGFR mutation detection

Positive and negative concordance between ARMS-PCR and SMART methods for detection of 29 hot spot EGFR mutations in tumour specimens was used to judge the performance of both assays (Table 2). Overall, there was a high degree of concordance, particularly for the most frequently detected mutations L858R and exon 19 deletions. In the 74 EGFR ARMS-PCR mutation-positive samples, SMART assay detected 71 positive samples but missed three with exon 19 deletions. In the remaining 110 samples that were negative for EGFR mutations by ARMS-PCR, 105 were also negative by SMART assay but five samples were positive for E20 insertion, E19 deletion and L858R mutations and two samples were positive for the T790M mutation.

**Table 2:** Concordance of SMART and ARMS-PCR assay for detection of EGFR mutations

EGFR mutations	ARMS-PCR assay	SMART assay		Performance of SMART assay	
		Positive	Negative	Sensitivity (95% CI)	Specificity (95% CI)
L858R	Positive (31)	31	0	100% (86.3%-100%)	99.3% (95.9%-100%)
	Negative (153)	1	152		
E19 del	Positive (41)	38	3	92.7% (79.0%-98.1%)	99.3% (95.6%-100%)
	Negative (143)	1	142		
T790M	Positive (2)	2	0	100% (19.8%-100%)	98.9% (95.7%-99.8%)
	Negative (182)	2	180		
E20 ins	Positive (0)	0	0	NC (NC)	99.5% (96.5%-100%)
	Negative (184)	1	183		
All	Positive (74)	71	3	95.9% (87.8%-98.9%)	95.5% (89.2%-98.3%)
	Negative (110)	5	105		
All	True positive (79)	78	1	98.7%* (92.2%-99.9%)	99.0%* (94.0%-100%)
	True negative (105)	1	104		

Sensitivity = 100% x (true positives/true positives + false negatives) Specificity = 100% x (true negatives/true negatives + false positives)

* Values based on true positive and negative samples. NC, not calculable

The basis of the false negative and false positive SMART assay results were further investigated (Supplementary Table S5). For the three “false negative” exon 19 del samples (CBR053, CBR070 and CBR012), Sanger sequencing of exon 19 PCR products derived from the original FFPE genomic DNA was performed as a secondary confirmation. For sample CBR053, Sanger sequencing confirmed the reference exon 19 sequence that was originally detected by SMART assay, indicating a false positive by ARMS-PCR. For sample CBR070, Sanger sequencing confirmed the presence an 18 bp insertion in exon 19(c.2214-2231) indicating a false negative by SMART assay. Similarly, for sample CBR012, Sanger sequencing confirmed the presence of a 15 bp deleted segment of exon19 (c.2235-2249), indicating a false negative exon 19 deletion by SMART assay.

Re-examination of the SMART assay data for the five “false positive” samples CBR008, CBR025, CBR098, CBR132 and CBR187 revealed very low mutation levels of 0.1% (E20 insertion), 0.12% (E19 deletion), 0.83% (T790M), 1.7% (T790M) and 3.13% (L858R), respectively (Supplementary Table S5). Given that the lower limit of detection sensitivity for ARMS-PCR is in the order of 1% [36], three samples fell below the detection threshold and two samples fell just above the detection threshold. In summary, benchmarking against the ARMS-PCR assay, the SMART assay had an overall detection sensitivity of 98.7% (78 out of 79 true positive samples) and a 99.0% specificity (104 out of 105 true negative samples).

### Clinico-pathogenic associations of EGFR mutations

Overall, the SMART assay detected a total of 85 EGFR mutations in 81 samples (Supplementary Table S2). In terms of mutation prevalence, the most frequent mutations were E19 deletions (42, 49.4%) and L858R (31, 36.5%), followed by T790M (4, 4.7%), E20 insertions (2, 2.4%), L861Q (3, 3.5%), G719A (2, 2.4%) and a novel exon 19 insertion (1.1%). By correlation of EGFR mutation status with the clinicopathological characteristics of the 184 NSCLC patients (Table 1), a significantly higher frequency of EGFR mutations were observed in females versus males (P < 0.05) and in non-smokers versus smokers (P < 0.05). There was no significant difference in the frequency of EGFR mutations in patients with adenocarcinoma (AC) or squamous carcinoma (SC) or, in patients with stage I, II, III, or IV disease. Of 34 stage IV patients identified with an activating EGFR mutation by SMART assay, 23 (67.6%) were subsequently treated with a regimen of TKIs (Supplementary Table S6). The median progression free survival (PFS) was 11 months, with a range of 1 to 24 months (Supplementary Figure S1). The Cox analysis showed that there was no correlation between EGFR activating mutation ratio and PFS (P=0.359, HR=0.407, 95% confidence interval 0.060-2.778).

### Spectrum of EGFR, KRAS, BRAF, TP53 and ALK mutations

**Figure 4:**
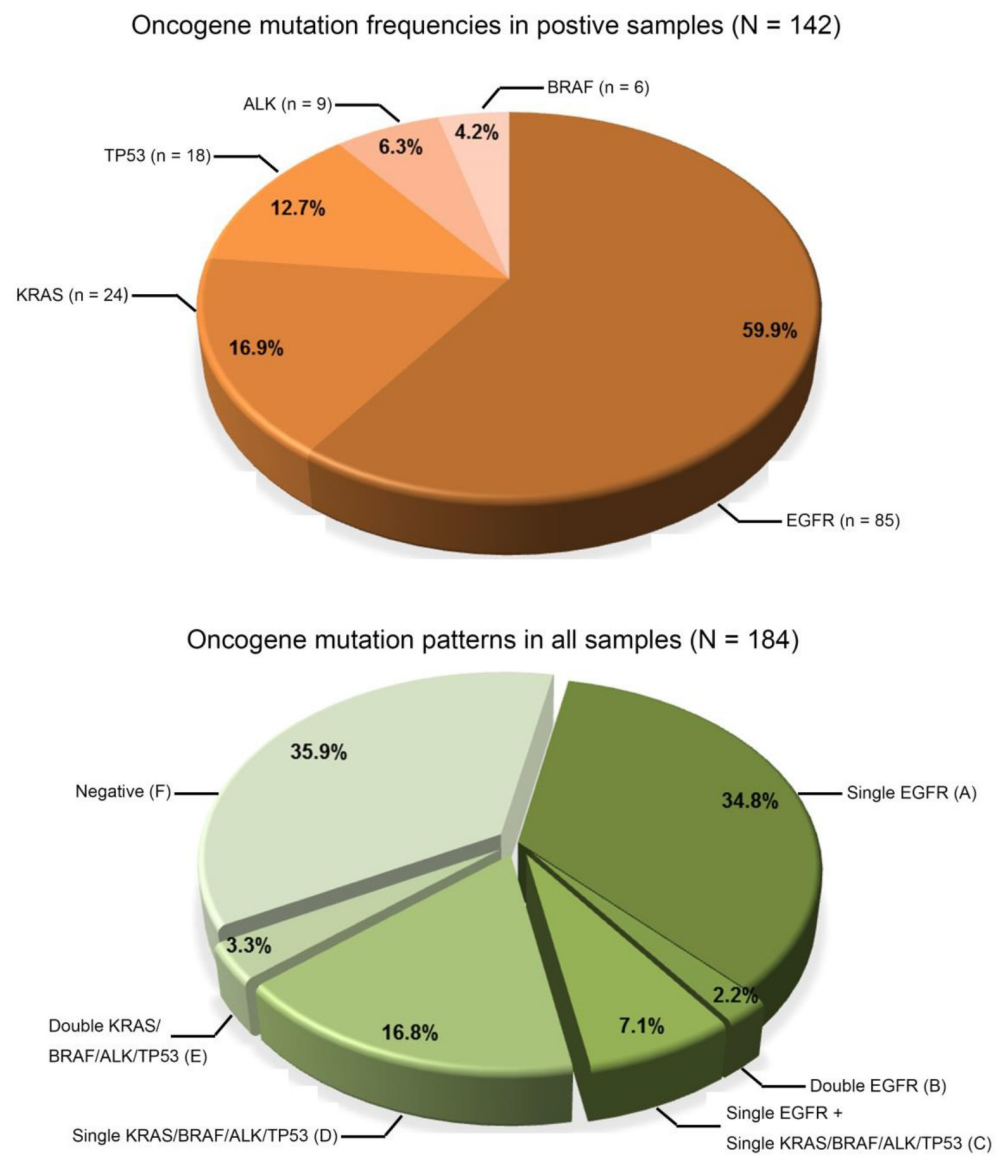
Spectrum of oncogenic mutations Pie charts show the frequency of mutations detected and the six different patterns of mutations (groups A-F) in the 184 NSCLC tumour specimens.

By SMART assay, a total of 142 mutations were detected in the 184 tumour specimens (Figure 4, Supplementary Table S2). EGFR mutations (85, 59.9%) were the most frequent followed by mutations in KRAS (24, 16.9%), TP53 (18, 12.7%), ALK (9, 6.3%) and BRAF (6, 4.2%) (Figure 4). The most frequent oncogenic variants found in EGFR, KRAS, BRAF, TP53 and ALK were, respectively, exon 19 deletions (49.4%), G12C/D/H/S/V mutations (76%), V600E mutations (100%), R248Q/W mutations (39%) and EML4 fusions (89%).

A variety of mutation patterns were identified in the 184 samples (Figure 4), including 64 (34.8%) with a single EGFR mutation (group A), 4 (2.2%) with two different EGFR mutations (group B), 13 (7.1%) with one EGFR mutation in coexistence with either a KRAS, BRAF, ALK or TP53 mutation (group C), 31 (16.8%) with either a single mutation in KRAS, BRAF, ALK or TP53 (group D), 6 (3.3%) with co-existing mutations in either the KRAS, BRAF, ALK or TP53 (group E) and 66 (35.9%) were negative (group F). Overall, 51.6% of samples (groups A and D) had a single mutation and 12.6% of samples (groups B, C and E) had two mutations. In all samples with two co-existing mutations, the mutation combinations and their respective levels were different.

**Figure 5:**
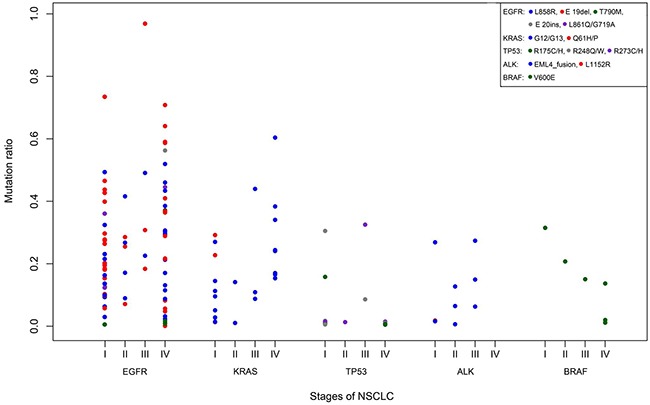
Nature and abundance of oncogenic mutations according to disease stage Gene mutations are coloured coded (legend).

There was no significant difference in the frequency and abundance of the different types of tumour EGFR, KRAS, TP53, BRAF and ALK fusions mutations according to stage of disease (Figure 5). Mutations levels varied widely from as low as 0.1% to as high as 96% and level ranges were also similar across disease stages. Adjusting for differences in patient numbers, drug sensitive EGFR mutations L858R and E19 deletions were similarly prevalent in stage I, II, III and IV patients. In addition, the eight potentially drug sensitive ALK fusions were found at all disease stages, except stage IV. Of the four T790M and two E20 insertion EGFR resistance mutations, three of the four T790M mutations and both E20 insertions were exclusively found in stage IV patients; the one exception was the remaining T790M mutation found in a stage I patient.

### Characterisation of EML4-ALK fusions

**Figure 6:**
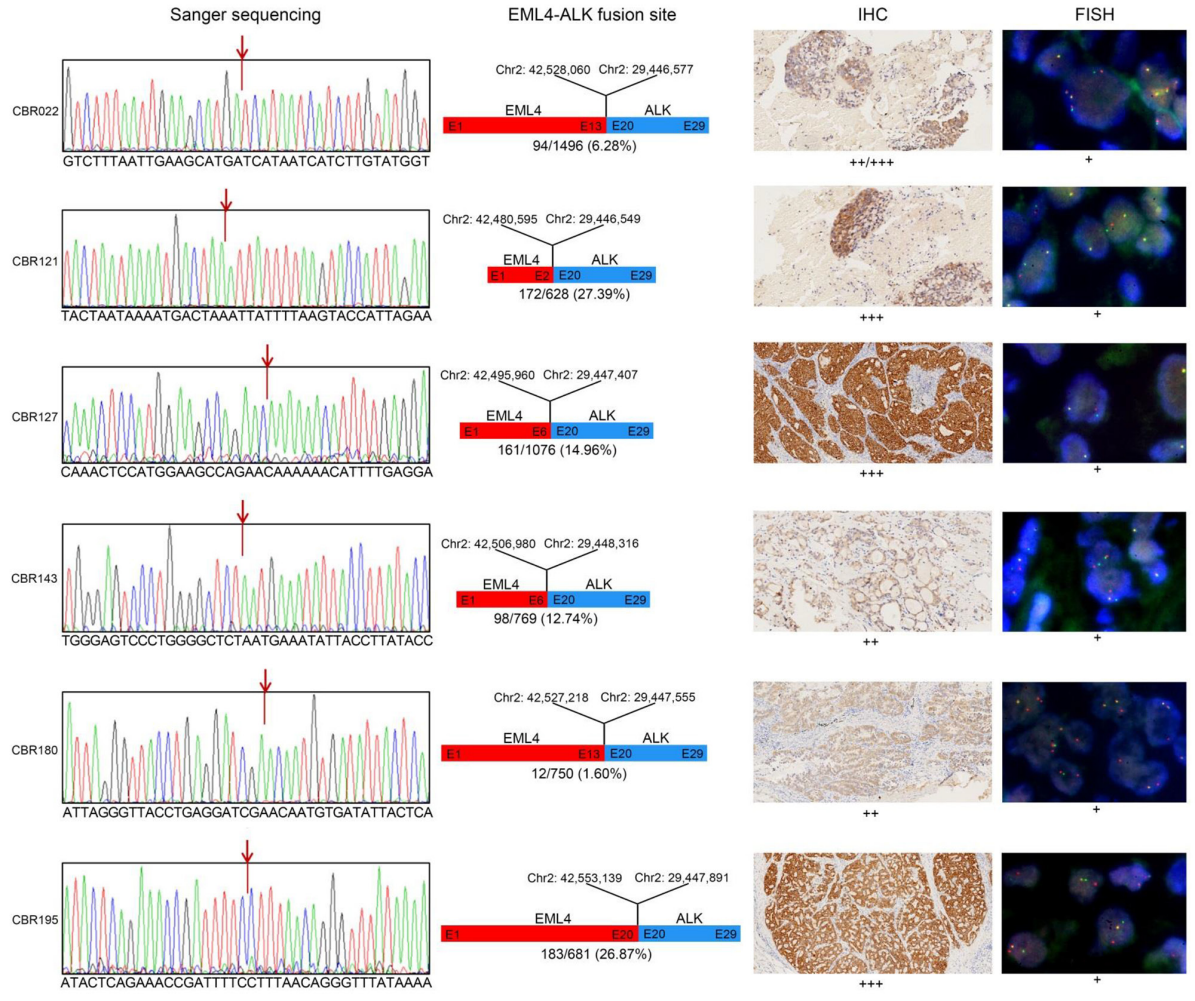
Molecular analysis of EML4-ALK fusion variants Panel 1. Sanger sequencing confirmed the fusion site between EML4 and ALK predicted from the paired end sequence reads generated by SMART assay. Panel 2. Diagrammatical representation of each fusion variant, showing the fusion site with respect to hg19 genome reference co-ordinates, EML4 and ALK exon (E) fusion positions and their tissue abundance. Panel 3. Level of EML4-ALK fusion protein detected by IHC in FFPE tissue, indicated by brown staining. Panel 4. In situ EML4-ALK DNA fusions are indicated by co-localization of orange and green FISH signals.

The SMART assay identified seven samples with EML4-ALK fusions and one sample with the ALK mutation L1152R at levels ranging from 0.65-27.39% (Supplementary Table S2). In five of the six ALK fusion positive samples, a single unique EML4-ALK fusion rearrangement was identified involving different exonic breakage points within the *EML4* gene and similar exon 20 breakage points within *ALK* gene (Figure 6). In the remaining sample (CBR143), two related EML4-ALK fusions varying in the exon 20 fusion site by 10 nucleotides were identified at different levels of 12.6% and 6.6% respectively.

To confirm the accuracy of the SMART assay for detecting and correctly mapping these fusion sites, we performed confirmatory Sanger sequencing of PCR products harbouring the fusion site derived from the original genomic DNA. For the five single ALK fusions and the one double ALK fusion, the determined breakpoint sequences were identical to those mapped by the SMART assay (Figure 6). As further confirmation, using the gold standard fluorescent in situ hybridization (FISH) assay for ALK fusions, all samples were positive for the ALK rearrangement (Figure 6). In addition, immunohistochemistry (IHC) analysis of tissue sections also showed positive ALK fusion staining of all samples, although staining intensity did not strongly correlate with predicted SMART assay levels of ALK fusion DNA fragments. For the double ALK fusion positive sample CBR143, there was no IHC evidence of two differentially staining populations of cells.

## DISCUSSION

In this study, a comprehensive survey of hot spot mutations in the *EGFR*, *KRAS*, *BRAF*, *TP53* and *ALK* genes in 184 NSCLC patients using the single allelic molecule counting SMART assay revealed highly heterogeneous tumour mutation profiles regardless of disease stage. The most prevalent mutations detected were activating EGFR mutations followed by KRAS, TP53, ALK and BRAF mutations. Over 50% of tumour tissue had a single mutation event with mutation loads ranging widely between patients at the same disease stage. Two independent mutation events were less commonly observed accounting for 12.6% of tumour tissue. These general trends observed in our unselected cohort of NSCLC patients are consistent with other oncogenic mutation studies focussed mainly on NSCLC patients with advanced disease [1, 7, 21, 27, 35]. Taken together, our findings support the large body of accumulated evidence that random mutation events are common in patients with NSCLC.

In modelling experiments, we demonstrated that the SMART assay was highly accurate for measuring levels of common EGFR variants across a wide dynamic range. As a benchmark against the gold standard ARMS-PCR assay, the SMART assay was highly sensitive and specific for detection of the 29 hot spot EGFR mutations. Although there were five false positives by SMART assay, all were below or at the threshold of detection for ARMS-PCR which is approximately 1% [32]. Since we showed in validation studies that the SMART assay can detect mutation levels down to 0.01%, the five “false positive” samples are likely to be true positives. However, large scale Sanger sequencing of cloned allelic fragments has not yet been undertaken on these five samples as a definitive confirmation. In contrast, two of the three “false negatives” exon 19 deletions were independently confirmed as true negatives. On this basis, re-adjustment of the concordance data for EGFR mutation detection by taking into account the all true positives and negatives, the sensitivity and specificity of the SMART assay could be as high as 98.7% and 99.0%, respectively.

In regard to T790M, this point mutation in exon 20 is the major resistance mechanism of EGFR-TKI with a frequency of around 50% in TKI resistant patients [12, 13, 36]. In contrast, in pre-treatment patients, the true frequency of T790M still remains controversial with rates in FPPE specimens varying from as low as 2% to as high as 40% depending on the type and sensitivity of the detection method employed [37–39]. In a recent study using a highly sensitive assay with a validated sensitivity of 0.1% [40], T790M mutations were detected in pre-TKI NSCLC patients at a rate of 2.8%. Similarly, in this study, we detected the T790M mutation by ARMS-PCR and SMART assay at rates of 1.1% and 2.2%, respectively. Of note, the two T790M positive samples missed by ARMS-PCR were found to have very low T790M levels of 0.83% and 1.7% by SMART assay.

As a quality control measure for the cSMART assay, we investigated whether activating mutations present in the tumour tissue could be detected in corresponding histological normal adjacent lung tissue. In all 25 cases investigated, no detectable activating mutation in tumor tissue was found in the control tissue, indicating a strong association of the mutation with tumourogenesis as well as high specificity of the cSMART assay for detection of these mutation types. Surprisingly, however, in four of the 25 adjacent non-tumour tissues samples, the assay also identified a different somatic mutation at very low levels, including two exon 20 insertion variants, one exon 19 deletion variant in EGFR and one R248W mutation in TP53 that were not detected in the original matching tumour sample. In these cases, the TP53 mutation may represent low-level somatic variants present in the surrounding tissue, resulting in relatively benign cell clones. For example, TP53 mutations specific to only the non-tumour lung tissue have been reported at an incidence of 12.5% [41]. However, in the cases of the new EGFR exon 19 deletion mutation and exon 20 insertion identified with very low abundance (0.26% for exon 19 deletion and 0.06%, and 0.04% for exon 20 insertion, respectively), this may represent an ongoing genetic evolution during the process of tumourogenesis in the cell clones of adjacent non-tumour tissue [42]. On the other hand, tumor heterogeneity may explain why these new identified mutations were detected in adjacent tissue but not in tumor tissue [43].

The study highlights several key advantages of the SMART assay over ARMS-PCR for detecting patient-specific oncogenic mutations in patients with NSCLC. Firstly, the multiplex design allowed surveying of 67 EGFR, KRAS, BRAF, TP53 and ALK hot spot mutations in a single assay format. In recent studies of different cohorts of NSCLC patients, a low frequency of NSCLC driver mutations in other oncoproteins have now been identified [21, 22, 27, 28]. Therefore, it should be possible to design additional primers to target the relevant hot spot mutations in these genes and develop an even more comprehensive SMART assay for NSCLC. Moreover, an expanded SMART assay could also be used as a discovery tool for revealing novel oncogenic mutations. Secondly the ability to quantitate the level of each mutation down to very low levels (one molecule per 1,000) provides valuable clinical information about the penetrance of the mutation in each tissue specimen and what proportion of tumour cells could be potentially targeted by drug therapies. Thirdly, with the emergence of promising reports showing the ability to detect EGFR mutations in the circulating plasma DNA of NSCLC patients [44], the SMART assay should serve as a useful diagnostic tool for broad oncogenic mutation surveillance of plasma as well as for accurately monitoring changes in the level of patient-specific mutations in response to targeted drug therapy.

In addition to the ability to detect point mutations and small deletions and insertions, the SMART assay also showed proof of concept for detection, quantitation and precise breakpoint identification of different EML4-ALK fusion variants and, may provide an alternative to recently developed PCR methods [32, 45]. Moreover, through the construction of the paired end allelic reads, the resulting DNA sequence allowed precise mapping of the fusion site and identified the nature of each EML4-ALK fusion variant. This additional diagnostic feature of the SMART assay will not only improve the diagnosis of EML4-ALK fusions in NSCLC, but potentially with clever primer design, will allow the development of new SMART assays for the specific detection and identification of other ALK fusion variants such as the NPM-ALK fusion associated with anaplastic large cell lymphomas [46], and obviate reliance on FISH and IHC as the gold standard techniques for detection of ALK fusions. As a corollary, the same general diagnostic approach using a suite of novel SMART kits could also be equally applied to target and detect clinically significant RET and ROS1 fusions that are associated with the disease progression of other types of lung cancers [47].

At the clinical level, the most significant observation was the presence of a high frequency and level of EGFR, KRAS, BRAF, ALK and TP53 mutations in stage I disease. In fact, the mutation profiles of stage I, II, III and IV patients were remarkably similar, with some samples in both groups displaying extremely high mutation loads. In a recent study of 637 stage I and II patients undergoing surgical resection [48], the pre-determined patterns for EGFR mutations closely resembled those found in our study cohort of 89 patients with early disease. These two studies add further support to the notion that activating mutations associated with NSCLC can arise very early in the evolution of tumourogenesis and act as the primary pathogenic drivers. The finding of a high mutation load of an activating mutation in early stage disease has potential clinical value for early diagnosis of lung cancer. Taking advantage of a recently developed SMART assay for circulating tumour DNA [44], it should also be possible to expand the multiplex panel of non-invasive DNA biomarkers further to serve as an adjuvant diagnostic tool for detection of early lung cancer.

## CONCLUSION

Using a novel SMART assay, we show that the spectrum of EGFR, KRAS, TP53, BRAF and ALK hot spot mutations in tumour specimens collected from early and advanced stage NSCLC patients are highly heterogeneous. With ability to now comprehensively survey oncogenic tumour mutations and identify activating and resistance mutations and their relative abundance using the SMART assay, mutation analysis will add significant clinical value to the management of NSCLC.

## MATERIALS AND METHODS

### Patients

The research study was approved by Human Ethics Committee of Hangzhou First People's Hospital (2015-28-01). A total of 184 unselected patients newly diagnosed with NSCLC at the Oncology Department of the Hangzhou First People's Hospital agreed to participate into the study. All patients gave written informed consent for specimen collection, provision of clinical information and biomarker analysis.

### Pathological analysis of tumour specimens

Tumour specimens collected either by surgery, fine needle aspiration or by bronchoscopy (> 2% of the total tissue mass and > 150 cells) were formalin-fixed and paraffin-embedded (FFPE) for histology. Histologic evaluation of stained FFPE tumour sections was performed independently by two experienced pathologists to confirm the diagnosis of NSCLC. Macro-dissection was performed to enrich the tumor tissue percentage to around 80% before DNA extraction. The criteria used for pathological assessment and clinical disease staging was according to the 2004 WHO classification guidelines and the TNM staging system of the IASLC (version 7), respectively.

### Preparation of genomic DNA from formalin fixed biopsy samples

Five to ten FFPE sections (5mm thickness) of each tumour specimen were used to prepare genomic DNA for mutation profiling by ARMS-PCR and SMART assays. Isolation of genomic DNA was performed using the AmoyDxÒ FPPE DNA Kit and DNA purification spin columns (Amoy, Xiamen, Fujian), according to the detailed protocol.

### EGFR mutation detection by ARMS-PCR assay

ARMS-PCR for detection of 29 hot spot EGFR mutations (Supplementary Table S1) was performed using the Human *EGFR* Gene Mutations Fluorescence Polymerase Chain Reaction (PCR) Diagnostic Kit (Amoy Diagnostics, Xiamen, Fujian) as previously described [32].

### EGFR, KRAS, BRAF, ALK and TP53 mutation detection by SMART assay

Counting of uniquely barcoded single allelic molecules in plasma to detect and quantitate the level of EGFR, KRAS, BRAF, TP53 and ALK mutations in tumour FFPE DNA was performed using a novel multiplex SMART assay, based on similar principles for fetal genotyping of plasma samples from pregnant women [34]. In brief, 50 ng of FFPE DNA was fragmented in NEB Next dsDNA fragmentase buffer (New England Biolabs, US) to an average size of 300 bp. DNA libraries were prepared as previously described [49] except that a degenerate 4 bp barcode sequence was incorporated into the sequencing adaptor for the purpose of uniquely identifying and counting single allelic molecules. Single DNA molecules were circularized and targeted with back-to-back primers located within 20-48 nucleotides of the mutant loci of interest.

A total of 28 targeting primer pairs were designed to simultaneously detect a total of 67 hot spot mutations in *EGFR* exons 18, 19, 20 and 21, *KRAS* exons 2, 3 and 4, *TP53* exons 4, 6 and 7, *BRAF* exon 15 and *ALK* exons 20, 22, 24 and 25 (Supplementary Table S1). For detection of ALK fusions, an additional 26 targeting primer pairs positioned sequentially along the 3 prime end of exon 19, intron 19 and the 5 prime end of exon 20 were also designed. The final multiplex SMART assay consisted of 54 targeting primer pairs. Following amplification of the targeted alleles by inverse PCR, paired end sequencing was performed on the MiSeq platform (Illumina). Based on overlapping paired end sequences of each molecule, single alleles were reconstructed using fastq-join software (http://code.google.com/p/ea-utils). A minimum of 1,000 unique allelic molecules were counted, and mutation levels of > 0.1% were defined as positive. The mutation ratio was calculated as the number of mutant alleles/total alleles.

### Confirmation of EML4-ALK fusions

EML4-ALK fusions were independently confirmed by FISH and IHC analysis of 4μM serial sections of FPPE fixed tissue. FISH was performed using the Vysis ALK Break Apart FISH Probe Kit (Abbott, Singapore) according to the supplier's protocol. For IHC, immunoreactive ALK antigen was retrieved by incubation in 0.01 mol/L citrate buffer solution (pH 6.0) at 100°C for 10 min. Endogenous peroxidase activity was inactivated by further incubation in 3% hydrogen peroxide for 10 min. ALK fusion protein was finally detected by incubation with anti-ALK (D5F3) rabbit MAb (Cell Signalling Technology, US) followed by a secondary mouse anti rabbit HRP-linked IgG antibody (Dako REAL™ EnVision™/HRP, Rabbit/Mouse detection system). ALK fusion protein staining was finally revealed using the DAB developing solution (Dako Cytomation, Glostrop, Denmark).

### Statistical methods

Comparison of expected and experimentally determined mutation levels was performed by Pearson's correlation. The relationship of EGFR gene mutations with clinical and pathological characteristics of the NSCLC patients was assessed by the Chi-squared test. PFS was estimated by the Kaplan-Meier method, and the relationship of EGFR activating mutation ratio and PFS was assessed by the Cox regression analysis. A P value of < 0.05 was considered statistically significant.

## SUPPLEMENTARY MATERIALS FIGURES AND TABLES






