# Supplementary material for: Comprehensive profiling and quantitation of oncogenic mutations in non small-cell lung carcinoma using single molecule amplification and re-sequencing technology
Source: Oncotarget. 2016 Jul 7;7(31):50477–89. doi: 10.18632/oncotarget.10464 (PMC5226597; doi:10.18632/oncotarget.10464)
Supplement: Supplementary file 1 [file oncotarget-07-50477-s001.pdf]

Comprehensive profiling and quantitation of oncogenic mutations in non small-cell lung carcinoma using single molecule amplification and re-sequencing technology

SUPPLEMENTARY FIGURE AND TABLES

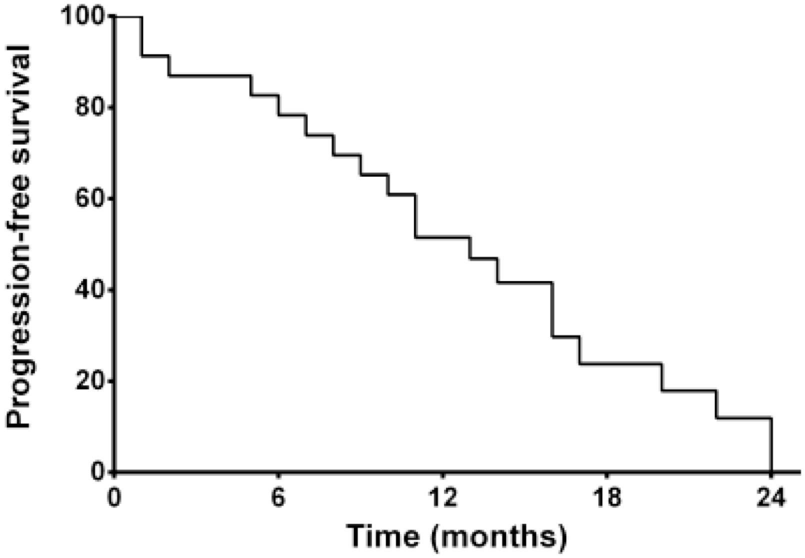

Supplementary Figure S1: Progression-free survival in 23 patients treated with TKIs.

Supplementary Table S1: Gene exons and hotspot mutations surveyed by the multiplex SMART assay

Gene	Exon	Hotspot mutations	Number of mutations	Inverse primer pairs
EGFR	18	G719S, G719C, G719A	3	2
	19	E746_A750del (COSM6223), E746_A750del (COSM6225), L747_T751del (COSM6254), L747_T751del (COSM12369), E746_T751>I, E746_T751del, E746_T751>A, E746_S752>A, E746_S752>V, E746_S752>D, L747_A750>P, L747_T751>Q, L747_E749del, L747_S752del, L747_A750>P, L747_A753>Q, L747_T751>P, L747_P753>S, L747_T751>S	19	2
	20	S768I, T790M, V769_D770insASV, D770_N771insG, H773_V774insH	5	2
	21	L858R, L861Q	2	2
KRAS	2	G12S, G12C, G12R, G12D, G12A, G12V, G13D	7	2
	3	Q61K, Q61E, Q61R, Q61L, Q61P, Q61H (COSM554), Q61H (COSM555)	7	2
	4	A146P, A146V, A146T	3	2
BRAF	15	V600E, V600D, V600K, V600R	4	2
ALK	20	Intron 19 – EML4 fusions, Exon 20 – EML4 fusions	1	26
	22	T1151Tins, L1152R, C1156Y, I1171N, F1174L	5	2
	24	L1196M, G1202R, D1203N, S1206Y	4	2
	25	G1269A	1	2
TP53	4	R175C, R175H	2	2
	6	R248W, R248Q	2	2
	7	R273C, R273H	2	2
**Total**			**67**	**54**

Supplementary Table S2: Oncogenic mutation profiles versus pathology and stage of NSCLC.

See Supplementary File 1

Supplementary Table S3: Tumour pathology and stage of NSCLC in mutation negative patients.

See Supplementary File 2

Supplementary Table S4: Oncogenic mutation profiles in matching tumour and non-tumour tissue

Sample number	Mutation gene	Mutation type	SMART assay result			
			Tumour specimen		Non tumour specimen	
			Mutation/total templates	Mutation ratio	Mutation/total templates	Mutation ratio
CBR017	EGFR	E746_A750del	446/2407	18.53%	0/3545	0
CBR018	EGFR	E746_A750del	26479/27332	96.88%	0/2749	0
CBR019	EGFR	E19 del exon19:c.2238_2252 del:p.746_751del	635/1549	40.99%	0/3151	0
CBR021	EGFR	L858R	234/1783	13.12%	0/3490	0
CBR024	EGFR	L858R	87/1344	6.47%	0/3341	0
CBR035	EGFR	E746_A750del	446/1604	27.81%	0/3120	0
CBR036	EGFR	E746_A750del	99/1391	7.12%	0/2807	0
CBR040	EGFR	L858R	1092/2626	41.58%	0/2280	0
CBR041	EGFR	L858R	393/2297	17.11%	0/2639	0
CBR046	ALK	fusion	5/767	0.65%	Not detected	-
CBR048	EGFR	L858R	50/1574	3.18%	0/3068	0
CBR057	EGFR	L858R	155/1634	9.49%	0/3420	0
	TP53	R248W	Not detected	-	63/3124	2.02%
CBR060	EGFR	E746_A750del	305/1658	18.40%	0/3108	0
	EGFR	E19del exon19:c.2236_2244 del:p.746_748del	Not detected	-	8/3110	0.26%
CBR071	EGFR	L858R	173/1931	8.96%	0/3423	0
CBR094	EGFR	E19 del exon19:c.2236_2244 del:p.746_748del	386/1975	19.54%	0/2439	0
CBR100	EGFR	L858R	197/872	22.59%	0/3643	0
CBR115	EGFR	L858R	1559/3177	49.07%	0/3787	0
	EGFR	E20ins c.2315_2316insCCACGT: p.P772delinsPHV	Not detected	-	2/3080	0.06%
CBR121	ALK	fusion	172/628	27.39%	Not detected	-
CBR124	EGFR	E746_A750del	418/1358	30.78%	0/3487	0
CBR127	ALK	fusion	161/1076	14.96%	Not detected	-
CBR128	EGFR	L858R	449/1384	32.44%	0/2769	0
CBR129	EGFR	E746_A750del	202/1112	18.17%	0/2958	0
CBR134	EGFR	L858R	293/1094	26.78%	0/4276	0
CBR135	EGFR	L858R	315/1554	20.27%	0/3242	0
	EGFR	E20ins exon20:c.2320_2321insT GC:p.V774delinsVL	Not detected	-	1/2249	0.04%
CBR136	EGFR	E19 del exon19:c.2238_2249 del:p.746_750del	259/1277	20.28%	0/2972	0

Supplementary Table S5: Summary of discordant EGFR mutation detection by ARMS-PCR and SMART assay

Sample number	ARMS-PCR assay result	SMART assay result			Sanger sequencing confirmation
		Mutation site	Mutation/ total templates	Mutation ratio	
CBR053	E19 del positive	E19 del	0/2014	0%	Normal exon 19 sequence (Exon 19 del negative)
CBR070	E19 del positive	E19 del	65/829	7.84%	c.2214_2215, insAAAATTCCCGTCGCTATC (Exon 19 del negative)
CBR112	E19 del positive	E19 del	0/2359	0%	c.2235_2249del15 (Exon 19 del positive)
CBR008	negative	E20 ins	1/979	0.10%	Not confirmed
CBR025	negative	E19 del	2/1653	0.12%	Not confirmed
CBR098	negative	T790M	10/1202	0.83%	Not confirmed
CBR132	negative	T790M	12/706	1.70%	Not confirmed
CBR187	negative	L858R	29/927	3.13%	Not confirmed

Table S6: Progression-free survival in patients treated with TKIs

Patient number	EGFR mutation	Mutation ratio	T790M mutation status	PFS
CBR019	E19 del: c.2238_2252del:p.746_751del	40.99%	-	13.00
CBR027	E19 del: L747_P753>S	70.81%	-	22.00
CBR039	E19 del: L747_P753>S	8.22%	-	14.00
CBR052	E19 del: L747_P753>S	59.04%	+	17.00
CBR075	E19 del: E746_A750del	64.06%	-	10.00
CBR081	E19 del: c.2250_2251del:p.750_751del	29.20%	-	13.00
	E19 del: c.2237_2246del:p.746_749del	29.45%		
CBR107	E19 del: c.2238_2252del:p.746_751del	36.85%	-	16.00
CBR118	E19 del: E746_A750del	4.75%	-	16.00
CBR139	E19 del: E746_A750del	28.87%	-	14.00
CBR066	E19 del: E746_A750del	1.09%	-	1.00
CBR014	L858R	51.94%	-	2.00
CBR021	L858R	13.12%	-	8.00
CBR048	L858R	3.18%	-	22.00
CBR061	L858R	46.03%	-	1.00
CBR077	L858R	21.27%	-	9.00
CBR085	L858R	2.36%	-	6.00
CBR091	L858R	38.51%	-	7.00
CBR093	L858R	43.39%	-	24.00
CBR105	L858R	30.43%	-	5.00
CBR125	L858R	29.67%	-	11.00
CBR126	L858R	17.05%	-	11.00
CBR138	L858R	8.77%	-	10.00
CBR079	L861Q	44.54%	-	20.00

PFS = Progression-free survival.
