# Supplementary material for: Comprehensive profiling and quantitation of oncogenic mutations in non small-cell lung carcinoma using single molecule amplification and re-sequencing technology
Source: Oncotarget. 2016 Jul 7;7(31):50477–89. doi: 10.18632/oncotarget.10464 (PMC5226597; doi:10.18632/oncotarget.10464)
Supplement: Supplementary file 2 [file oncotarget-07-50477-s002.docx]

| **Sample number** | **Sample information** | | | **SMART assay results** | | | |
| --- | --- | --- | --- | --- | --- | --- | --- |
|  | **Sample collected from primary or metastatasis tumor** | **Histology** | **Stage** | **Mutant gene** | **Mutation** | **Mutation/**  **total templates** | **Mutation ratio** |
| **Single EGFR mutations** | | | | | | | |
| CBR014^#^ | Metastasis tumor biopsy | AC | IV | EGFR | L858R | 1462/2815 | 51.94% |
| CBR021^#^ | Primary tumor biopsy | AC | IV | EGFR | L858R | 234/1783 | 13.12% |
| CBR024 | Primary tumor tissue | AC | I | EGFR | L858R | 87/1344 | 6.47% |
| CBR040 | Primary tumor tissue | AC | II | EGFR | L858R | 1092/2626 | 41.58% |
| CBR041 | Primary tumor tissue | AC | II | EGFR | L858R | 393/2297 | 17.11% |
| CBR048^#^ | Primary tumor biopsy | AC | IV | EGFR | L858R | 50/1574 | 3.18% |
| CBR057 | Primary tumor tissue | AC | I | EGFR | L858R | 155/1634 | 9.49% |
| CBR061^#^ | Primary tumor biopsy | AC | IV | EGFR | L858R | 824/1790 | 46.03% |
| CBR071 | Primary tumor tissue | AC | II | EGFR | L858R | 173/1931 | 8.96% |
| CBR077^#^ | Metastasis tumor biopsy | AC | IV | EGFR | L858R | 224/1053 | 21.27% |
| CBR085^#^ | Primary tumor biopsy | AC | IV | EGFR | L858R | 42/1781 | 2.36% |
| CBR091^#^ | Primary tumor biopsy | AC | IV | EGFR | L858R | 880/2285 | 38.51% |
| CBR093^#^ | Primary tumor biopsy | AC | IV | EGFR | L858R | 663/1528 | 43.39% |
| CBR100 | Metastasis lymph nodes biopsy | SC | III | EGFR | L858R | 197/872 | 22.59% |
| CBR103 | Primary tumor biopsy | AC | IV | EGFR | L858R | 331/1092 | 30.31% |
| CBR105^#^ | Primary tumor biopsy | AC | IV | EGFR | L858R | 399/1311 | 30.43% |
| CBR115 | Metastasis lymph nodes biopsy | AC | III | EGFR | L858R | 1559/3177 | 49.07% |
| CBR125^#^ | Metastasis tumor biopsy | AC | IV | EGFR | L858R | 273/920 | 29.67% |
| CBR126^#^ | Primary tumor biopsy | AC | IV | EGFR | L858R | 155/909 | 17.05% |
| CBR128 | Primary tumor tissue | AC | I | EGFR | L858R | 449/1384 | 32.44% |
| CBR134 | Primary tumor tissue | AC | II | EGFR | L858R | 293/1094 | 26.78% |
| CBR135 | Primary tumor tissue | AC | I | EGFR | L858R | 315/1554 | 20.27% |
| CBR138^#^ | Primary tumor biopsy | AC | IV | EGFR | L858R | 103/1174 | 8.77% |
| CBR172 | Primary tumor tissue | AC | I | EGFR | L858R | 186/860 | 21.63% |
| CBR181 | Primary tumor tissue | AC | I | EGFR | L858R | 94/683 | 13.76% |
| CBR187 | Primary tumor tissue | AC | I | EGFR | L858R | 29/927 | 3.13% |
| CBR015 | Primary tumor biopsy | AC | IV | EGFR | E19 del: L747_P753>S | 77/1362 | 5.65% |
| CBR017 | Primary tumor tissue | AC | I | EGFR | E19 del: E746_A750del | 446/2407 | 18.53% |
| CBR018 | Metastasis lymph nodes biopsy | AC | III | EGFR | E19 del: E746_A750del | 26479/27332 | 96.88% |
| CBR019^#^ | Primary tumor biopsy | AC | IV | EGFR | E19 del: c.2238_2252del:p.746_751del | 635/1549 | 40.99% |
| CBR025 | Primary tumor biopsy | SC | IV | EGFR | E19 del: L747_P753>S | 2/1653 | 0.12% |
| CBR027^#^ | Primary tumor biopsy | AC | IV | EGFR | E19 del: L747_P753>S | 2988/4220 | 70.81% |
| CBR035 | Primary tumor tissue | SC | I | EGFR | E19 del: E746_A750del | 446/1604 | 27.81% |
| CBR036 | Primary tumor tissue | AC | II | EGFR | E19 del: E746_A750del | 99/1391 | 7.12% |
| CBR039^#^ | Primary tumor biopsy | AC | IV | EGFR | E19 del: L747_P753>S | 127/1545 | 8.22% |
| CBR043 | Primary tumor tissue | AC | I | EGFR | E19 del: L747_P753>S | 98/211 | 46.45% |
| CBR058 | Primary tumor tissue | AC | I | EGFR | E19 del: c.2250_2273del:p.750_758del | 170/572 | 29.72% |
| CBR060 | Metastasis lymph nodes biopsy | AC | III | EGFR | E19 del: E746_A750del | 305/1658 | 18.40% |
| CBR065 | Primary tumor biopsy | AC | IV | EGFR | E19 del: E746_A750del | 688/1681 | 40.93% |
| CBR067 | Primary tumor tissue | AC | II | EGFR | E19 del: c.2236_2244del:p.746_748del | 322/1263 | 25.49% |
| CBR068 | Primary tumor biopsy | AC | IV | EGFR | E19 del: c.2238_2252del:p.746_751del | 259/1194 | 21.69% |
| CBR069 | Primary tumor biopsy | AC | IV | EGFR | E19 del: E746_A750del | 478/1313 | 36.41% |
| CBR075^#^ | Primary tumor biopsy | AC | IV | EGFR | E19 del: E746_A750del | 2007/3133 | 64.06% |
| CBR078 | Primary tumor biopsy | AC | IV | EGFR | E19 del: L747_P753>S | 756/2057 | 36.75% |
| CBR082 | Primary tumor tissue | AC | I | EGFR | E19 del: c.2236_2244del:p.746_748del | 196/1985 | 9.87% |
| CBR094 | Primary tumor tissue | AC | I | EGFR | E19 del: c.2236_2244del:p.746_748del | 386/1975 | 19.54% |
| CBR107^#^ | Primary tumor biopsy | AC | IV | EGFR | E19 del: c.2238_2252del:p.746_751del | 723/1962 | 36.85% |
| CBR108 | Primary tumor biopsy | SC | IV | EGFR | E19 del: c.2250_2273del:p.750_758del | 477/2217 | 21.52% |
| CBR118^#^ | Metastasis tumor biopsy | AC | IV | EGFR | E19 del: E746_A750del | 28/590 | 4.75% |
| CBR124 | Metastasis tumor biopsy | AC | III | EGFR | E19 del: E746_A750del | 418/1358 | 30.78% |
| CBR129 | Primary tumor tissue | AC | I | EGFR | E19 del: E746_A750del | 202/1112 | 18.17% |
| CBR131 | Metastasis lymph nodes biopsy | AC | IV | EGFR | E19 del: E746_A750del | 1196/2038 | 58.68% |
| CBR136 | Primary tumor tissue | AC | I | EGFR | E19 del: c.2238_2249del:p.746_750del | 259/1277 | 20.28% |
| CBR139^#^ | Primary tumor biopsy | AC | IV | EGFR | E19 del: E746_A750del | 347/1202 | 28.87% |
| CBR173 | Primary tumor tissue | AC | I | EGFR | E19 del: E746_A750del | 223/810 | 27.53% |
| CBR176 | Primary tumor tissue | AC | I | EGFR | E19 del: E746_A750del | 1641/2240 | 73.26% |
| CBR182 | Primary tumor tissue | AC | I | EGFR | E19 del: E746_A750del | 418/980 | 42.65% |
| CBR189 | Primary tumor tissue | AC | I | EGFR | E19 del: E746_A750del | 75/721 | 10.40% |
| CBR190 | Primary tumor tissue | AC | I | EGFR | E19 del: E746_A750del | 600/1375 | 43.64% |
| CBR132 | Primary tumor biopsy | AC | IV | EGFR | T790M | 12/706 | 1.70% |
| CBR008 | Primary tumor biopsy | AC | IV | EGFR | E20ins:c.2319_2320insGCT:p.H773delinsHA | 1/979 | 0.10% |
| CBR079^#^ | Metastasis tumor biopsy | AC | IV | EGFR | L861Q | 918/2061 | 44.54% |
| CBR092 | Primary tumor tissue | SC | I | EGFR | G719A | 301/1522 | 19.78% |
| CBR099 | Primary tumor biopsy | SC | IV | EGFR | E20ins:c.2311_2312insACCCCC:p.N771delinsNPH | 1120/1990 | 56.28% |
| **Double EGFR mutations** | | | | | | |  |
| CBR081^#^ | Primary tumor biopsy | AC | IV | EGFR | E19 del: c.2250_2251del:p.750_751del | 483/1654 | 29.20% |
|  |  |  |  | EGFR | E19 del: c.2237_2246del:p.746_749del | 483/1640 | 29.45% |
| CBR052^#^ | Metastasis tumor biopsy | AC | IV | EGFR | E19 del: L747_P753>S | 1630/2761 | 59.04% |
|  |  |  |  | EGFR | T790M | 796/2144 | 37.13% |
| CBR175 | Primary tumor tissue | AC | I | EGFR | E19 del: E746_A750del | 359/1360 | 26.40% |
|  |  |  |  | EGFR | T790M | 10/1317 | 0.76% |
| CBR050 | Primary tumor tissue | AC | I | EGFR | L861Q | 281/2049 | 13.71% |
|  |  |  |  | EGFR | G719A | 330/2642 | 12.49% |
| **Single EGFR + single KRAS/BRAF/ALK/TP53** | | | | | | | |
| CBR051 | Metastasis tumor biopsy | AC | IV | EGFR | L858R | 506/4397 | 11.51% |
|  |  |  |  | TP53 | R175H | 5/981 | 0.51% |
| CBR095 | Primary tumor tissue | AC | I | EGFR | L858R | 461/1989 | 23.18% |
|  |  |  |  | TP53 | R273H | 16/1310 | 1.22% |
| CBR109 | Primary tumor biopsy | AC | IV | EGFR | L858R | 478/1559 | 30.66% |
|  |  |  |  | TP53 | R248W | 13/1169 | 1.11% |
| CBR191 | Primary tumor tissue | AC | I | EGFR | L858R | 111/675 | 16.44% |
|  |  |  |  | TP53 | R175C | 9/1327 | 0.68% |
| CBR198 | Primary tumor tissue | AC | I | EGFR | L858R | 95/944 | 10.06% |
|  |  |  |  | TP53 | R248W | 6/1021 | 0.59% |
| CBR066^#^ | Primary tumor biopsy | AC | IV | EGFR | E19 del: E746_A750del | 12/1104 | 1.09% |
|  |  |  |  | KRAS | G12C | 300/1764 | 17.01% |
| CBR178 | Primary tumor tissue | AC | I | EGFR | E19 del: E746_A750del | 46/780 | 5.90% |
|  |  |  |  | KRAS | Q61P | 4/305 | 1.31% |
| CBR013 | Primary tumor tissue | AC | II | EGFR | E19 del: E746_A750del | 131/459 | 28.54% |
|  |  |  |  | TP53 | R273H | 4/303 | 1.32% |
| CBR104 | Primary tumor tissue | AC | I | EGFR | E19 del: E746_A750del | 284/713 | 39.83% |
|  |  |  |  | TP53 | R248Q | 362/1186 | 30.52% |
| CBR188 | Primary tumor tissue | AC | I | EGFR | E19 del: E746_A750del | 69/446 | 15.47% |
|  |  |  |  | TP53 | R273C | 9/595 | 1.51% |
| CBR070 | Primary tumor tissue | SC | I | EGFR | E19: c.2214_2215 insAAAATTCCCGTCGCTATC | 65/829 | 7.84% |
|  |  |  |  | TP53 | R175C | 11/1229 | 0.90% |
|  |  |  |  | TP53 | R248Q | 14/815 | 1.72% |
| CBR098 | Primary tumor biopsy | SC | IV | EGFR | T790M | 10/1202 | 0.83% |
|  |  |  |  | TP53 | R273H | 8/539 | 1.48% |
| CBR177 | Primary tumor tissue | AC | I | EGFR | L861Q | 441/1223 | 36.06% |
|  |  |  |  | ALK | L1152R | 14/762 | 1.84% |
| **Single KRAS/BRAF/ALK/TP53 mutations** | | | | | | | |
| CBR011 | Primary tumor tissue | AC | I | KRAS | G12C | 37/723 | 5.12% |
| CBR016 | Primary tumor biopsy | AC | IV | KRAS | G12C | 2745/4544 | 60.41% |
| CBR026 | Primary tumor tissue | AC | I | KRAS | G12D | 53/1876 | 2.83% |
| CBR034 | Primary tumor tissue | AC | II | KRAS | G12C | 190/1343 | 14.15% |
| CBR037 | Primary tumor biopsy | AC | IV | KRAS | G12C | 241/988 | 24.39% |
| CBR059 | Metastasis lymph nodes biopsy | AC | III | KRAS | G12D | 22/202 | 10.89% |
| CBR063 | Primary tumor tissue | AC | I | KRAS | Q61H | 155/681 | 22.76% |
| CBR076 | Metastasis tumor biopsy | AC | IV | KRAS | G12D | 342/1421 | 24.07% |
| CBR083 | Primary tumor tissue | AC | I | KRAS | G12D | 193/1330 | 14.51% |
| CBR088 | Primary tumor tissue | AC | I | KRAS | G12A | 144/1506 | 9.56% |
| CBR110 | Metastasis lymph nodes biopsy | SC | IV | KRAS | G12C | 193/1257 | 15.35% |
| CBR144 | Metastasis lymph nodes biopsy | MC | III | KRAS | G12V | 83/944 | 8.79% |
| CBR155 | Metastasis lymph nodes biopsy | AC | IV | KRAS | G12V | 595/1552 | 38.34% |
| CBR156 | Metastasis lymph nodes biopsy | AC | III | KRAS | G13D | 453/1030 | 43.98% |
| CBR157 | Primary tumor biopsy | AC | IV | KRAS | G12V | 295/866 | 34.06% |
| CBR159 | Primary tumor biopsy | AC | IV | KRAS | G12D | 118/713 | 16.55% |
| CBR185 | Primary tumor tissue | AC | I | KRAS | G12S | 125/463 | 27.00% |
| CBR074 | Metastasis lymph nodes biopsy | AC | IV | BRAF | V600E | 37/1853 | 2.00% |
| CBR087 | Primary tumor biopsy | AC | IV | BRAF | V600E | 26/2236 | 1.16% |
| CBR112 | Primary tumor tissue | AC | I | BRAF | V600E | 532/1689 | 31.50% |
| CBR145 | Metastasis lymph nodes biopsy | AC | III | BRAF | V600E | 226/1502 | 15.05% |
| CBR164 | Primary tumor biopsy | AC | IV | BRAF | V600E | 138/1008 | 13.69% |
| CBR022 | Metastasis lymph nodes biopsy | AC | III | ALK | EML4-ALK fusion | 94/1496 | 6.28% |
| CBR046 | Primary tumor tissue | AC | II | ALK | EML4-ALK fusion | 5/767 | 0.65% |
| CBR121 | Metastasis lymph nodes biopsy | AC | III | ALK | EML4-ALK fusion | 172/628 | 27.39% |
| CBR127 | Metastasis lymph nodes biopsy | AC | III | ALK | EML4-ALK fusion | 161/1076 | 14.96% |
| CBR180 | Primary tumor tissue | AC | I | ALK | EML4-ALK fusion | 12/750 | 1.60% |
| CBR086 | Primary tumor biopsy | AC | IV | TP53 | R248W | 20/2091 | 0.96% |
| CBR130 | Primary tumor biopsy | AC | IV | TP53 | R175H | 7/960 | 0.73% |
| CBR140 | Metastasis lymph nodes biopsy | AC | III | TP53 | R248Q | 104/1209 | 8.60% |
| CBR193 | Primary tumor tissue | AC | III | TP53 | R273C | 258/793 | 32.53% |
| **Double KRAS/BRAF/ALK/TP53 mutations** | | | | | | | |
| CBR186 | Primary tumor tissue | AC | I | KRAS | G12C | 12/425 | 2.82% |
|  |  |  |  | KRAS | G13D | 6/410 | 1.46% |
| CBR149 | Primary tumor tissue | AC | II | KRAS | G12D | 10/943 | 1.06% |
|  |  |  |  | BRAF | V600E | 175/844 | 20.73% |
| CBR142 | Primary tumor tissue | AC | I | KRAS | G12D | 34/301 | 11.30% |
|  |  |  |  | TP53 | R175H | 289/1827 | 15.82% |
| CBR097 | Primary tumor tissue | AC | I | KRAS | Q61H | 440/1507 | 29.20% |
|  |  |  |  | TP53 | R248W | 11/1788 | 0.62% |
| CBR143 | Primary tumor tissue | AC | II | ALK | EML4-ALK fusion | 98/769 | 12.74% |
|  |  |  |  | ALK | EML4-ALK fusion | 51/788 | 6.47% |
| CBR195 | Primary tumor tissue | AC | I | ALK | EML4-ALK fusion | 183/681 | 26.87% |
|  |  |  |  | TP53 | R175H | 4/657 | 0.61% |

Legend: AC = adenocarcinoma; SC = squamous carcinoma; MC = mucoepidermoid carcinoma

^#^ TKI treatment following diagnosis
