# Supplementary material for: Comprehensive profiling and quantitation of oncogenic mutations in non small-cell lung carcinoma using single molecule amplification and re-sequencing technology
Source: Oncotarget. 2016 Jul 7;7(31):50477–89. doi: 10.18632/oncotarget.10464 (PMC5226597; doi:10.18632/oncotarget.10464)
Supplement: Supplementary file 3 [file oncotarget-07-50477-s003.docx]

| **Sample number** | **Sample collected from primary or metastatasis tumor** | **Histology** | **Stage** |
| --- | --- | --- | --- |
| CBR006 | Primary tumor tissue | AC | I |
| CBR010 | Metastasis tumor biopsy | NC | IV |
| CBR012 | Primary tumor tissue | AC | I |
| CBR020 | Metastasis lymph nodes biopsy | SC | III |
| CBR023 | Primary tumor tissue | AC | I |
| CBR028 | Primary tumor biopsy | AC | IV |
| CBR029 | Metastasis lymph nodes biopsy | AC | III |
| CBR030 | Primary tumor tissue | AC | I |
| CBR031 | Metastasis tumor biopsy | AC | IV |
| CBR032 | Primary tumor tissue | AC | I |
| CBR042 | Metastasis lymph nodes biopsy | AC | III |
| CBR044 | Primary tumor tissue | SC | I |
| CBR045 | Primary tumor tissue | AC | II |
| CBR047 | Primary tumor tissue | AC | I |
| CBR049 | Primary tumor tissue | SC | II |
| CBR053 | Metastasis tumor biopsy | AC | IV |
| CBR054 | Metastasis tumor biopsy | AC | IV |
| CBR055 | Primary tumor tissue | SC | I |
| CBR056 | Primary tumor tissue | AC | II |
| CBR064 | Metastasis lymph nodes biopsy | AC | III |
| CBR072 | Metastasis tumor biopsy | AC | IV |
| CBR073 | Primary tumor tissue | AC | II |
| CBR080 | Metastasis lymph nodes biopsy | AC | III |
| CBR084 | Primary tumor tissue | AC | II |
| CBR089 | Primary tumor tissue | AC | II |
| CBR090 | Primary tumor tissue | AC | II |
| CBR096 | Primary tumor biopsy | AC | IV |
| CBR102 | Primary tumor tissue | NC | I |
| CBR106 | Primary tumor biopsy | SC | IV |
| CBR111 | Primary tumor tissue | AC | I |
| CBR116 | Metastasis tumor biopsy | SC | IV |
| CBR117 | Primary tumor biopsy | SC | IV |
| CBR120 | Primary tumor biopsy | AC | IV |
| CBR122 | Metastasis tumor biopsy | AC | III |
| CBR123 | Primary tumor biopsy | AC | IV |
| CBR133 | Primary tumor biopsy | SC | IV |
| CBR137 | Primary tumor tissue | AC | II |
| CBR141 | Primary tumor tissue | AC | I |
| CBR146 | Primary tumor tissue | AC | II |
| CBR147 | Primary tumor tissue | AC | II |
| CBR148 | Metastasis lymph nodes biopsy | AC | III |
| CBR150 | Primary tumor tissue | AC | II |
| CBR151 | Primary tumor tissue | AC | I |
| CBR152 | Primary tumor biopsy | SC | IV |
| CBR153 | Primary tumor tissue | SC | II |
| CBR154 | Primary tumor tissue | AC | I |
| CBR158 | Primary tumor biopsy | AC | IV |
| CBR160 | Metastasis lymph nodes biopsy | AC | III |
| CBR161 | Metastasis tumor biopsy | AC | IV |
| CBR162 | Primary tumor biopsy | AC | IV |
| CBR163 | Primary tumor biopsy | AC | III |
| CBR165 | Primary tumor tissue | AC | I |
| CBR166 | Primary tumor biopsy | AC | IV |
| CBR167 | Metastasis lymph nodes biopsy | SC | III |
| CBR168 | Metastasis lymph nodes biopsy | AC | III |
| CBR169 | Primary tumor tissue | SC | I |
| CBR170 | Metastasis lymph nodes biopsy | AC | III |
| CBR171 | Primary tumor tissue | AC | III |
| CBR174 | Primary tumor tissue | SC | I |
| CBR179 | Primary tumor tissue | MC | I |
| CBR183 | Primary tumor tissue | SC | II |
| CBR184 | Primary tumor tissue | AC | I |
| CBR192 | Primary tumor tissue | AC | II |
| CBR196 | Primary tumor tissue | SC | II |
| CBR197 | Primary tumor tissue | AC | I |

Legend: AC = adenocarcinoma; SC = squamous carcinoma; NC = neuroendocrine carcinoma;

MC = mucoepidermoid carcinoma.
